# Ten Recommendations for US Programs Hosting Global Health Partners

**DOI:** 10.5334/aogh.4699

**Published:** 2025-09-09

**Authors:** Ana Maria Crawford, Michelle Arteaga, Rodrigo Rubio, Gaston Nyirigira, Samy Bendjemil, James C. Hudspeth, Tracy L. Rabin

**Affiliations:** 1 Clinical Professor, Department of Anesthesiology, Perioperative and Pain Medicine, Stanford University, Stanford, CA, USA; 2 Global Engagement Programs Manager, Department of Anesthesiology, Perioperative and Pain Medicine, Stanford University, Stanford, CA, USA; 3 Consultant Anesthesiologist, Development Centre Director, Centro Médico ABC, Mexico City, Mexico; 4 Senior Consultant Anesthesiologist and Lecturer, Department of Anesthesia and Critical Care, King Faisal Hospital Rwanda, Africa Health Sciences University, Kigali, Rwanda; 5 Clinical Fellow, Department of Surgery, Stanford University, Stanford, CA, USA; 6 Clinical Associate Professor, Department of Medicine, Boston University, Boston, USA; 7 Associate Professor of Medicine, Department of Internal Medicine, Yale School of Medicine, New Haven, CT, USA

**Keywords:** global health, global exchange, professional development, international medical graduates, bidirectional partnership, hosting, training

## Abstract

*Background:* US academic institutions increasingly seek to engage in global health education through bidirectional partnerships. One innovative approach is hosting International Medical Graduates (IMGs) at US-based programs, offering short-term global health learning experiences locally while expanding professional opportunities for IMGs from both high-resource and resource-constrained settings.

*Methods:* Drawing on over 15 years of collective experience, this paper brings together perspectives from global authors to identify practical strategies for hosting visiting IMGs. The recommendations address the operational, legal, and financial barriers that institutions often face when building such programs.

*Recommendations:* Key challenges include securing stakeholder engagement, navigating visa limitations, and addressing funding and institutional policy gaps. The authors outline 10 actionable recommendations designed to guide US institutions in building ethical, sustainable, and mutually beneficial IMG-hosting programs.

*Conclusion:* While prior literature has emphasized the ethical foundations and benefits of global partnerships, this paper offers concrete guidance to support the development of IMG-hosting initiatives. These programs advance the goals of equity, reciprocity, and long-term partnership in global health education.

## Introduction

Global health aspires to achieve the highest attainable standard of health for all people. A critical component of strong healthcare systems is a well-supported workforce, yet across much of the globe, there are too few physicians due to limited opportunities for education, training, and professional development [1–3]. At the same time, many clinicians and trainees in highly resourced settings, such as the USA, seek meaningful opportunities for global engagement [4]. As demand grows across US academic institutions for global learning experiences, one distinct strategy is to host international physician colleagues at US programs.

In particular, hosting International Medical Graduates (IMGs)—a regulatory term used by the Educational Commission for Foreign Medical Graduates (ECFMG) to refer to individuals who received their medical degree outside the USA and Canada—offers a practical mechanism to support bidirectional learning and strengthen long-term global partnerships [5]. Hosting, in this context, involves inviting IMGs to participate in observerships, short-term academic experiences, or education-focused collaborations within US institutions. Creating these exchange programs promotes global health learning within US programs while expanding opportunities for IMGs, including those from both highly resourced and resource-constrained practice settings [5].

Hosting IMGs provides bidirectional benefits for trainees and faculty alike, and for both hosts and visitors. International visitors facilitate US trainees’ exposure to case-based discussions of unique disease states, a greater understanding of diverse patient populations, greater intercultural cognizance, analysis of practice differences and a global context relevant to healthcare systems [5]. These interactions also enhance faculty development, prompting reflective dialogue about cost-conscious care, frugal innovation, sustainable practices, and culturally nuanced communication. Such benefits align with broader efforts to internationalize medical education and promote cultural humility among healthcare professionals [6]. Moreover, when US institutions host colleagues from resource-constrained settings, US personnel gain insights into how care is delivered under different constraints, prompting deeper appreciation for adaptable strategies and resilience [7].

Reciprocally, US-based hosting programs offer visiting physicians opportunities for professional development, advanced training, and exposure to alternative healthcare systems. IMGs from lower resourced settings are often hindered by the lack of exposure to specialty and subspecialty training [1, 3]. Hosting such colleagues in highly resourced settings allows them to experience an alternative healthcare system and subsequently decide for themselves which processes could be most impactful if applied in their own systems.

Visiting IMGs also observe important similarities in clinical practice. For those working in resource-constrained settings, this reinforces the standards they have been taught and validates their own approaches. Recognizing these similarities allows them to shift their attention to more nuanced aspects of healthcare systems, identifying quality improvements applicable to their own contexts. By experiencing systems, processes, and professional culture first-hand, rather than only through didactic teaching, these providers are better equipped to evaluate and implement interventions in their home settings. Some resource-constrained providers come back validated by their experience, and some return better equipped to lead change in their local system.

Bidirectional programs bring balance to partnerships. Hosting programs can help dismantle power asymmetries by prioritizing co-creation of learning experiences, shared authorship, and mutual capacity building [8]. Bidirectionality in global health partnerships emphasizes mutual benefit grounded in shared learning, respect, and reciprocity, and is critical to ethical global health work [9]. Hosting programs that embrace this principle may see increased institutional capacity, improved trainee engagement, and more durable international collaborations [10, 11]. Yet, logistical challenges are considerable. Common challenges in building such programs include relevant stakeholder engagement, buy-in from leadership, visa and licensing challenges, and financial systems and funding among others. To facilitate a shift toward bidirectionality in global health education programs, this overview includes perspectives from authors from across the globe who have engaged in bidirectional programs for over 15 years. Here, 10 recommendations are outlined for building capacity to host IMGs in short-term, supervised training experiences at US institutions, with a focus on observership programs (Table 1). For clarity, IMGs participating in global exchange programs are referred to as “visiting observers” throughout.

**Table 1:** Ten recommendations for hosting IMGs within US institutions.

Recommendation 1: Gain Institutional Support by Demonstrating the Added Value of Hosting
Recommendation 2: Develop a Programmatic Checklist and Administrative Timeline
Recommendation 3: Become Familiar with Visa Types, Restrictions, and Allowable Roles for Visitors
Recommendation 4: Understand State Medical Licensing Restrictions for IMGs
Recommendation 5: Understand Tax Liabilities for Host Programs and Visiting Observers
Recommendation 6: Securing Programmatic Funding and Creating a Budget
Recommendation 7: Understand Institutional Requirements for Visitors
Recommendation 8: Ensure Adequate Program Administrative Support
Recommendation 9: Take Steps to Protect Visiting Observers in Emergencies
Recommendation 10: Adequately Consider Cultural and System Differences

## Recommendation 1: Gain Institutional Support by Demonstrating the Added Value of Hosting

We believe hosting foreign colleagues undoubtedly benefits host programs (Table 2). Yet, given the financial and administrative support these programs require, the first step in program development requires demonstration of this added value to institutional leadership. For many institutions, the value of global health work stems from opportunities for their own trainees and research interests for faculty [10, 11]. However, there are other direct benefits gained from hosting IMGs. Foreign colleagues bring with them a wealth of knowledge and unique experiences informing the practices of otherwise highly resourced colleagues. Those with resource constraints are masterful, yet frugal, innovators. These colleagues bring especially valuable lessons in resource utilization and adaptability to supply chain disruptions—growing concerns for US-based practices. Hosting talented colleagues also contributes to academic diversity, facilitating representation that is able to counter biased assumptions that resource constraints are analogous to constraints in knowledge, ability, or scholarship. Additionally, with unique cultural perspectives, international colleagues can inform the care provided for our own increasingly diverse patient populations found in US healthcare settings.

**Table 2:** Benefits of hosting foreign colleagues.

No Travel Required: Creates a local Global Health opportunity
Satisfaction: Facilitates recruitment, retention, and satisfaction of trainees and faculty
Well-being: Creates global community and philanthropic opportunities
Professional Development: Expands global networks and global engagement
Scholarship: Promotions and advancement through international collaborations
Global Preeminence: Global recognition for all partner institutions
Culturally Informed Care: Understanding diverse patient populations
Systems-Based Practice: Greater understanding of various healthcare systems, complexities, and unique solutions
Equity: Stimulates innovation in addressing health barriers and constraints
Patient Advocacy: Globalizes the reach of patient advocacy
Practice-Based Improvements: Facilitate adaptations to resource constraints and supply chain disruptions
Environmental Health: Advances “green” solutions, smart resource utilization, decreased environmental impact
International Health and Diplomacy: Heightens awareness of complex international health issues; greater understanding of stakeholders and governance

Ethical global engagement requires that partnerships be genuinely bidirectional, responding to ongoing calls for the decolonization of global health [12]. Current best practices emphasize that non-US partners must receive clear and tangible benefits [8, 11, 12]. Exchange programs offer a powerful mechanism to advance the goals of all participating institutions. Global exchange programs are international networking opportunities, leading to collaborations in research, quality improvement projects, international presentations, and publications.

## Recommendation 2: Develop a Programmatic Checklist and Administrative Timeline

Hosting foreign colleagues requires thoughtful preparation and extensive consideration. To ensure compliance with local, state, and federal requirements, and to legally safeguard visitors, explicit programmatic descriptions and intentions must be prospectively considered during program design. Logistical considerations, including visa requirements, medical licensure, financial systems, tax implications, budgeting, patient privacy, program administration, and numerous cultural considerations, take time to navigate. Understanding requirements at the federal, state, and institutional levels is imperative. Programmatic checklists can facilitate administrative processes (Table 3) and should follow a timeline considerate of pre-arrival preparations, tasks for optimizing hosting, and post-departure feedback and evaluations.

**Table 3:** Example checklist for hosting IMG visiting observers (VOs).

**Ongoing Advocacy**	
□	Advocate for bidirectional programs as mission, vision, values; Highlight value of academic and workforce recruitment; Framing relevant to local, state, national, and international initiatives and policies, such as World Health Organization Sustainable Development Goals
□	Foster global engagement with colleagues from other countries and diverse practice settings at educational events, professional networking events, and conferences
□	Join working groups to advocate for standardizing access for hands-on clinical education across countries and (US) states (e.g., Consortium of Universities for Global Health, professional society committees on legislative affairs)
**9–12 months in advance**	
□	Secure commitment from stakeholders including international offices, department chair, division chief, fellow faculty members
□	Determine length of stay for VOs to create the budget and confirm funding
□	Confirm buy-in from staff management and assigned administrative support
□	Verify VO approval process at the institution/hospital and any other clinical locations across the health system; Identify managers in each clinical unit for approval as necessary
**6–9 months in advance**	
□	Establish selection criteria and application process for VOs; Create an application form, selection committee, and scoring rubric; Publicize the opportunity across appropriate academic networks and partners or open to public; Promote on websites and social media
□	Send selection emails to VOs with program details, initial requirements for visa application, and steps involved in observing within the program
□	Confirm payment method to be used for program-related transactions; Consider an institutional credit card, or purchase orders; Reimbursement may be an acceptable payment method but not recommended, whenever possible
**3 months in advance**	
□	Confirm visa has been obtained
□	Begin health clearance process with individual visitors
□	Assess VOs’ specific clinical and academic interests and coordinate specialty hosts
□	Buy plane tickets and reserve lodging (local housing, Airbnb, dormitories, or hotel)
□	Create or update an orientation manual
□	National conference registration (optional, but recommended)
**4–6 weeks in advance**	
□	Create hosting and social schedule (including phone numbers and email list)
□	Research and buy health insurance policy, if needed
□	Plan a welcome dinner (especially for those staying for more than a couple of weeks)
□	Create evaluation forms
□	Create combined schedule of lectures (didactics, grand rounds, university lectures, etc.); can utilize shared calendar (e.g., Google Calendar or Outlook)
**1 week in advance of arrival**	
□	Health clearance and visiting observer paperwork signed off for each location and approved; ready for security badge
□	Food plan is ready (cafeteria dining plans or vouchers, grocery shopping or delivery, pre-paid credit cards)
□	Cell phone plan or loaner phones ready (institutional plan vs. external)
□	Ground transportation plan is ready (host pick-up, rideshare, other airport transportation)
□	Online collaborative workspace set up for sharing documents and collecting feedback (e.g., Google Drive)
**First day/week**	
□	Receive badge
□	Hospital orientation - dress codes, scrubs, locker room, hosting schedule and protocol; review workplace cultural differences
□	Campus and neighborhood tour (public transportation, grocery shopping, cell phone activation, laundry)
□	Set up rideshare account (i.e., Uber or Lyft, some institutions may have business Uber accounts)
□	Outline expectations for VO’s (e.g., write a daily or weekly report)
□	Host welcome dinner or event
**During stay**	
□	Assign team member for daily or frequent check-ins, review weekly reports
□	Set up informational meetings with institutional leaders and colleagues with similar interests and goals
□	Invite to present at and attend departmental and organizational lectures and events
□	Invite for social activities in the evenings and on the weekends (site seeing, sports games, happy hours, dinners, worship services)
□	Begin writing impact report for reporting back to leadership
**After departure**	
□	Work with finance to process financial transactions related to visit (documentation related to program should be collected before and during stay)
□	Send evaluation forms and surveys on impact and how to improve program
□	Follow up on ideas for collaboration (research, conference submission, QI project, future virtual and in-person exchange of knowledge)
□	Inspire other internal and external programs to host foreign medical graduates for short-term clinical education through storytelling and presenting data
□	Send notes of gratitude and impact report to leadership and administrators supporting the program

Additionally, programs must ensure smooth communication across institutional departments, such as graduate medical education offices, occupational medicine, security offices, and clinical departments. As such, it is of the utmost importance to have clearly defined roles for visitors. These roles can range from observing, to providing education, to learning, to clinical participation. Each type of role may have restrictions at the institutional, state, or federal levels. Financial and tax rules also impact allowable roles for visitors and are discussed in more detail in recommendations 3 and 5 below. Preparations must begin along appropriate timelines, considerate of the visitors’ ability to meet requirements and processing capacity. Resource-constrained settings often need additional time due to different financial systems, various approvals needed, language barriers, and limitations in internet access and bandwidth.

## Recommendation 3: Become Familiar with Visa Types, Restrictions, and Allowable Roles for Visitors

### US visa type and processes

Among the various visa types, the B-1 (business)/B-2 (visitor) and J-1 (exchange visitor) visas are the most commonly used for short-term, non-immigrant academic visits [13, 14]. For observerships, we recommend the B-visa option.

From a logistical standpoint, observership programs structured under the B-1 visa are the most straightforward to implement. The B-visa can be valid for up to 10 years, though individual stays are limited to six months. B-1 visas are self-sponsored and avoid the two-year home-country return requirement often associated with J-1 visas. However, B-1 visa holders cannot receive academic credit, compensation, or employment while in the USA. These restrictions influence how exchange programs are funded and the roles available to participants. Institutions must also use precise titles, as terms such as “fellow,” “scholar,” or “researcher” may imply employment or formal training, creating risks of visa non-compliance. These visa-related financial restrictions impact how exchange program hosts structure their funding of these initiatives (see below) and the various roles in which B-visa visitors can participate. Institutions must take care to label these visitors accurately.

Unfortunately, B-visa refusals are common, particularly for applicants from certain regions. The U.S. Department of State tracks refusal rates by country. At the time of writing, the average B-visa refusal rates were approximately 35% for Africa, 31% for the Middle East, 29% for Asia, and 27% for Latin America—compared to only 16% for Europe [15]. These refusals are often rooted in concerns about illegal immigration. Additionally, the US government is currently piloting visa bonds for certain countries due to concerns about overstays, even though these countries are not the primary source of visa overstays [16].

An informal, unpublished interview conducted by the authors with US Embassy staff in Rwanda in 2018 identified five factors that significantly influence visa decisions (see Table 4). Determining factors are largely reflective of the visitors’ ties to their home country, including financial, professional, and family ties that would deter visitors from emigration. Thus, observership programs should provide visitors with a letter to support their B-1 visa application which emphasizes the capacity-building nature of the visit and the expectation that the visit will result in contributions to medical education and patient care in the home country.

**Table 4:** Preparation for visa interviews.

FACTOR	REASONING	PROPOSED SOLUTIONS
Financial	Applicants must have sufficient funds to cover expenses for travel, especially the return flight, and their needs while in the USA.	Make clear in invitation letters plans to provide funding for the visiting physicians while in the USA, especially the return flight Clearly identify the person(s) responsible for overseeing the visitor while in the country Applicants MUST have a clear understanding of their funding and will be asked about it during the interviews Applicants should have itemized budgets from their hosts and take them to visa interview as a reference to present to embassy staff
Immigration Status	Applicants are non-immigrants and must prove strong ties to their home country and compelling reasons why they will return home instead of immigrating to the USA.	Demonstrate ties to home country: family (spouses and children), other business, other investments, important jobs/training, additional obligations such as military service Applicants must formulate good responses explaining reasons they plan to return home
Purpose of Visit	Applicants must demonstrate fluent knowledge of the program in which they are participating in the USA.	Host provides program descriptions directly to the visiting physicians prior to interview Applicants review carefully the institutional program descriptions including objectives and expectations and prepare to describe specifically what they will be doing in the USA for all dates Name the supervisor responsible for the visiting physician at each institution or site
**Purpose of Visit Example:** “I will spend 6 weeks as a visiting observer at Stanford Hospital in California to learn more about their healthcare system. I will also be teaching Stanford healthcare providers about healthcare in my setting. I will be spending each day observing their clinical and training programs. Through this partnership, and learning exchange, we will enhance training, improve professional skills, and improve the care for patients.”		
Length of Stay	Applicants must fully understand itineraries and recite where they will be on which dates while in the USA.	Each institution outlines in detail visitors’ itineraries and provide a copy of the schedule prior to the applicant’s interview Applicants should know the city, state, and address of their lodging
Prior Travel	It is viewed favorably if applicants have previous traveled outside of their home continent with demonstrated return to home.	Applicants should list any travel as evidence that they have left, but have also chosen to return

For those considering a J-1 visa pathway for short-term training experiences, options include the J-1 *Short-Term Scholar* or the J-1 *Research Scholar* visas. These visas do allow for supervised “incidental” patient contact; however the academic institution sponsor must provide a statement to certify that the focus of the program is observation, consultation, teaching, or research [13, 14]. Importantly, the J-1 “*Alien” Physician* visa used for formal graduate medical education or training requires additional certification by the ECFMG and sponsorship, a process which is inappropriate for short-term training due to the length and cost of the steps involved. J-1 visa holders may remain in the USA for 12 months to five years, depending on the specific program and visa type [13, 14]. In contrast to the B-visas, J-1 visas also require that hosts provide insurance coverage for medical care, medical evacuation, and repatriation of remains.

There are other important distinctions between the B- and J-1 visa categories, particularly regarding permitted activities for physicians. B-visa holders, including IMGs, are strictly limited to observation. They may not engage in direct patient care or hands-on clinical work. While observation is permitted under both B and J-1 visas, only J-1 visas allow limited, supervised clinical involvement when designated appropriately.

## Recommendation 4: Understand State Medical Licensing Restrictions for IMGs

### Understanding state licensing restrictions

Resource-constrained health systems often depend on international partners with higher resources to support healthcare workforce training. For example, Rwanda’s ambitious 4 × 4 program aims to quadruple their healthcare workforce in four years, the success of which partially depends upon its highly resourced partners to host and train providers [17]. Although short-term, supervised, hands-on clinical training experiences are feasible in Canada, as well as many other parts of the world, the visa issues described above are further compounded by state-level medical licensing regulations [18]. Many US states lack medical license categories appropriate for short-term clinical experiences for IMGs or restrict these opportunities only to IMGs with academic appointments or with exceptional talent or rare skill sets [18, 19].

To date, 18 states have established, or are considering, pathways to short-term practice and licensure for IMGs [20]. Advocacy efforts are underway to address state-level medical licensing barriers led by academic institutions, the Consortium of Universities for Global Health, and professional societies such as the American College of Surgeons [21]. Easing medical licensing restrictions for supervised, short-term training experiences, in addition to addressing visa challenges, could support burgeoning training programs in other countries, such as Rwanda’s 4x4 program, who hope to send their trainees abroad for specialty and sub-subspecialty training opportunities [17].

## Recommendation 5: Understand Tax Liabilities for Host Programs and Visiting Observers

### Understanding funding mechanisms and tax liability

US hosting programs will need funding for these types of exchange programs, as visiting IMGs require support for lodging, daily expenses, and travel. Funding sources and an example budget are provided in the next recommendation. Remembering that VOs are on a B-visa, they cannot be paid a salary or stipend, and observation programs should not be described as fellowships, employment, education for credit, scholarships, or research programs [13, 14]. These designations have different visa and funding implications. However, directly paid expenses and reimbursements are allowable. Although some visiting colleagues have funding, those from resource-constrained settings may not be able to self-fund. Where feasible, host programs should categorize the funding they provide as business expenses. This avoids imposing a tax liability upon their guests. Consultation with institutional tax experts is encouraged to ensure IRS compliance.

Paid honoraria are possible, and B-visa holders are eligible for honoraria, but not if their stay exceeds nine days or they have received five previous honoraria within the previous six months. This is referred to as the 9/5/6 rule [22]. B-visa holders are not eligible for social security numbers, and although expenses paid by another are not taxable, any payment that is considered income is taxable [23].

Therefore, programs must be aware of the tax implications for the visitor and the program if stipends, salaries, or other forms of payment are offered beyond what can be considered business expenses. Gaining institutional support that understands the added value of hosting (*Recommendation 1*) facilitates justification for funding and categorization of business expenses for these programs. Funds can also be sent directly to the partner program abroad, with the intent that they then reimburse the faculty directly. This can have the benefit of strengthening financial ties to the trusted partner program and normalizing the flow of funds to a partner as the effector. Processing time and associated fees should be considered for any financial transaction.

## Recommendation 6: Secure Programmatic Funding and Create a Budget

### Demonstrating funding needs

Although the cost of hosting is typically similar to the cost of travel for US faculty or trainees, it requires an institutional commitment to global health. Rarely are VOs from resource-constrained settings able to self-fund visits, whereas wealthier visitors may be able to pay administrative fees and pay for their own expenses. Ethical funding structures should be considered to address these disparities. Processes for offsetting costs for lower income VOs, tiered fee schedules, and creation of bursaries should be explored. We believe hosting IMG colleagues has a comparable if not greater return on investment as it increases global health exposure and engagement for more people within the hosting department.

### Funding sources

Sources of funding include departmental or institutional unrestricted funds, grants, industry partners, foundations, and individual philanthropists. Funding must remain ethical and transparent. Creating an endowment, when possible, can ensure sustainability of funding. Observers from wealthier countries often pay administrative fees to universities in order to visit and observe [24]. Another approach toward equitable engagement is to use administrative fees from wealthier visitors to help support funding for those from lower income countries or with greater demonstrated needs.

### Creating the budget

Funding for flights, lodging, ground transportation, and meals should be included. Costs can vary widely by region (Table 5). Using homestays or campus dormitories can alleviate high housing costs. Additional considerations are the registration fee and travel costs for any conferences included.

**Table 5:** Example budget-visiting observer (B-Visa), Palo Alto, California.

EXPENSES (1 VISITOR/ 1 MONTH)	NOTES	COST (BEST ESTIMATES)
Flights	Economy class	$2000
Lodging for 1 month	Shared if more than 1 person	$3000
Visa fee reimbursement	If approved	$185
Conference registration	Low-income country discounts available	$215
Ground transportation	Car hiring services or Taxis	$400
Travel/Health insurance	Emergency/Evacuation only	$300
Health clearance	Vaccines or Titers	$300
Meals	Consider as business meals	$2250
Phone + service	Pre-paid with loaner phone	$200
**Total**		**$8850**

### Salary and benefits considerations

Travelers, especially those on B-visas, must understand whether their salary and benefits will be continued by their home institution while they are away. Reimbursement programs for lost salary and benefits are not possible for B-visa holders, requiring the exploration of J-1 or other visa types. Any payment of stipends or honoraria to visitors is governed by visa and tax laws that must be fully understood by the host institution to ensure compliance. Health and evacuation insurance is a serious consideration for hosts and visitors alike. Whereas health insurance is not a federal requirement for hosts of B-visa holders, J-1 visa holders must be provided insurance for health and repatriation of remains by their US host employer.

## Recommendation 7: Understand Institutional Requirements for Visitors

### Occupational health services and clearance

Visitors must meet occupational health services’ requirements prior to observing or working in clinical areas. Obtaining visitors’ vaccination history and comparing required vaccines should be done prior to arrival. Some vaccines are not available or required in other countries, necessitating vaccination or titer checks upon arrival. Programs must ensure adequate time, funding, and consent for additional vaccinations before finalizing travel. While awaiting clearance, visitors may still engage in didactics, simulations, social activities, and other non-clinical experiences.

### Privacy and security compliance

Clearance from privacy and security offices must be sought from the hosting institution. Additional training such as HIPAA and workplace initiatives should be provided as necessary and may be done online in advance of travel. Visitors require badge access and clearance for all sites through which they will rotate. Patient consent to the presence of VOs is paramount. At teaching institutions this consent is often included in other general consent forms, whereas individual patient consent may be necessary in other settings. Inclusion within general consent forms is encouraged where feasible and ethical. As is standard with any learner, VOs should be introduced as such to each patient.

## Recommendation 8: Ensure Adequate Program Administrative Support

### Program administration and planning

Visiting observers need a daily schedule, location assignments, and contact persons throughout their rotation. Travel and visa planning is an extensive pre-arrival process, during which time program administrators can work to develop the daily schedules for the VOs. Visitors often desire exposure across various clinical subspecialties and hospital departments, which requires coordination. Similarly, faculty and trainees within the host institution often wish to engage with international visitors, necessitating introductions and further coordination. In addition to a primary point of contact, several staff, faculty, and trainees can be recruited as liaisons in order to spare the majority of the workload falling to only few people. Ample time for professional networking, observation, learning, and academic exchange is ideal. Needs assessments and evaluations are useful for informing the schedules of both the program and the visitor and should be arranged prior to arrival, during the program, and post-rotation.

## Recommendation 9: Take Steps to Protect Visiting Observers in Emergencies

### Medical and evacuation insurance

Although only the J-1 visa requires it, hosts of B-visa holders should explore and consider short-term emergency medical and evacuation coverage for their guests, especially for those who lack insurance coverage from their home countries. Physicians from low-resource settings are often the financial security for their immediate and extended family. Ensuring the least financial and medical liability is of utmost importance. Clear and frequent communication is necessary, including emergency contact information, needed medications, environmental, food or medication allergies, and other medical essentials. Incurring medical debt in the USA can expose visitors to financial hardship and jeopardize any future US travel and opportunities.

## Recommendation 10: Consider Cultural and System Differences to Optimize Logistics

### Language barriers

English proficiency is desirable; however, for many global visitors English is a second or third language. It is recommended that language and communications are clear and simple without complex instructions. Avoid the use of abbreviations, acronyms, and jargon, or provide a glossary of frequently used terms and abbreviations as a reference. Additionally, avoid brand names and be aware that names of medications can vary across settings. Consider translation services where feasible. Even if unnecessary on a moment-to-moment basis, a check-in periodically in a first language can be very helpful.

### Financial systems vary significantly

Many global colleagues do not have access to financial systems common in the USA, including personal credit cards or electronic payment applications. This cannot be over-stressed. It is often not feasible to have guests purchase their own flights or lodging. Further, reimbursements by check are not possible if travelers lack US bank accounts, and therefore, require wire transfers or cash equivalent. Institutional online payment portals may be difficult to navigate, and administrative assistance should be offered whenever possible. Wire transfers take days or weeks to process and are associated with fees for both the sender and recipient. Host programs should make every attempt to pay directly for expenses where feasible.

### Mobile phones and communication

During travel and while in the USA, global visitors may be dependent upon free Wi-Fi to access communication applications such as *Signal* or *WhatsApp*. Travelers may not have international phone plans. Access to communication methods should be arranged as needed before and during their visit. Although most people have phones, even unlocked internationally purchased cell phones may not function with locally purchased SIM cards. Consideration of loaner phones or local phones may be necessary. Embedded SIM (e-SIMs) are becoming more prevalent.

### Digital access

Global colleagues may not have continuous access to the internet, free access, or adequate bandwidth to download large amounts of data. Consideration of data limitations and digital literacy must be considered. Extra time may be necessary for global colleagues to respond to communications due to different time zones and cultural differences in response times. Explicitly state deadlines and expected response times. Avoid burdening colleagues with large amounts of costly data downloads. Consider the use of online forms as much as possible to avoid printing and scanning costs.

### Travel and ground transportation

Many people do not have global travel experience. Provide travel instructions assuming no prior experience with travel, airports, ground transportation, or international mobile phone access. Similarly, visitors may not be familiar with ground transportation or ride-sharing applications such as *Uber* or *Lyft*, both of which require accounts and credit cards. Institutional accounts are helpful in arranging transportation for visitors. Gift cards for these services may also have limitations such as requirements for a US bank account, rendering them less feasible. Hosts should provide information and instructions to facilitate public transportation access. Some institutions may offer free campus shuttles. Logistics should be streamlined and tested prior to visitors’ arrivals. Bicycles are not common in every culture, making this an option only for some. Prior to departure, ensure each visitor clearly understands travel options and instructions.

### Local orientation and opportunities

A comprehensive orientation is essential to help IMG visitors navigate and adapt to the new environment. A well-structured local orientation can significantly enhance the experience, safety, and effectiveness of visiting clinicians and learners. Orientation should begin with a campus tour, including key clinical areas, educational spaces, libraries, and support services. Attention to basic needs is equally important, providing guidance on transportation (e.g., public transit options, rideshare use, bike rentals), housing resources, dining facilities, and nearby shopping can greatly ease daily logistics. Safety should be a priority, and orientations should include practical information on personal and community safety, including how to respond to emergencies and access care if needed. Visitors will also benefit from culturally sensitive information about navigating social norms, local customs, and institutional expectations. Resources to support spiritual and cultural belonging are important. Offering information about nearby religious institutions, community organizations, and international student or visitor support groups can help IMG visitors feel more connected and welcomed. Clear guidance at the outset sets a respectful tone and ensures both visitor and institutional expectations are aligned.

Further, having visiting IMGs engage with grand rounds, academic programs, and relevant professional networks offers valuable opportunities for professional development, cross-cultural exchange, and deeper integration into the academic community—enhancing both their learning experience and the institution’s global perspective.

## Concluding Thoughts

Academic programs seeking to expand their global health programs must align their mission, vision, and values with initiatives that are most impactful for training globally informed physicians, while addressing global inequalities. These authors believe that hosting foreign colleagues is of benefit whether they hail from high-income countries or significantly constrained practice settings. Care of our patients is strengthened by exploring diverse practices and perspectives, including that of other healthcare systems and cultures. Ethical engagement in global health requires us to consider bidirectionality a requirement, not a discretionary courtesy. Hosting programs for global health comes with many challenges and considerations, but all are surmountable. Although there are many reasons to support IMG physicians visiting US programs, the most important are the improvements in culturally informed patient care and increased equity in professional development.

Even with bidirectional focus during development of hosting programs, there remain many barriers to reaching true equity for IMGs. Due to licensing and visa challenges, visiting colleagues are most often confined to observation only. To train or practice clinically in the USA, even for the short-term, requires navigation of complex processes at the state, federal, and institutional levels. With limits preventing participation in short-term clinical care, true exchange and learning are hindered. Advocates have formed working groups to address the barriers at all levels. Further, many US faculty, trainees, and students want to engage in global health initiatives but are unable to travel or take time away from work and family. Hosting IMG colleagues at US institutions offers a unique opportunity to create local global health opportunities that support US training program goals. International exchange facilitates US trainees to strengthen all their core competencies and serves to enrich the professional development of faculty and staff. The biggest challenge lies in the will of academic institutions.

## Confirmation Statements

We confirm that this manuscript has not been published and is not under consideration elsewhere. All tables and figures are created by the authors and are not reprinted. All authors had access to and participated in the preparation of this manuscript. Dr. Ana Maria Crawford and Michelle Arteaga contributed significantly to this manuscript including the creation of all 10 recommendations and the creation of all tables and figures based on their decade-long experience hosting IMGs at their institution. Dr. Rodrigo Rubio and Dr. Gaston Nyirigira both contributed significantly to the content of this paper and offered their unique and valuable perspectives as non-US practicing physicians who have participated in foreign exchange programs. Dr. Samy Bendjemil contributed significantly to research and in content creation regarding the US visa processes and rejection rates by geography. Dr. James Hudspeth and Dr. Tracy Rabin contributed significantly to the content of this paper using their expertise in global health, visa processes, and state licensure restrictions.
